# Protocol for *in vitro* transcribing mRNAs with defined poly(A)-tail lengths and visualizing sequential PABP binding

**DOI:** 10.1016/j.xpro.2024.103284

**Published:** 2024-08-31

**Authors:** Carmen Grandi, Martin Emmaneel, Frank H.T. Nelissen, Maike M.K. Hansen

**Affiliations:** 1 Institute for Molecules and Materials, Radboud University, Heyendaalseweg 135, 6525 AJ Nijmegen, the Netherlands; 2 Oncode Institute, Nijmegen, the Netherlands

**Keywords:** Bioinformatics, Cell-based Assays, Molecular Biology, Gene Expression

## Abstract

Quantifying the number of proteins that interact with mRNAs, in particular with poly(A) tails of mRNAs, is crucial for understanding gene regulation. Biochemical assays offer significant advantages for this purpose. Here, we present a protocol for synthesizing mRNAs with accurate, length-specific poly(A) tails through a PCR-based approach. We also describe steps for an *in vitro* (i.e., cell-free) approach for visualizing the sequential binding of Cytoplasmic Poly(A)-Binding Proteins (PABPCs) to these poly(A) tails. We detail quality control steps throughout the procedure.

For complete details on the use and execution of this protocol, please refer to Grandi et al.^1^

## Highlights

• *In vitro* synthesis of mRNAs with defined poly(A)-tail length
• Quality control procedures for both poly(A)-tailed PCR and mRNA products
• *In vitro* protocol for the visualization of PABPCs’ binding to the poly(A) tail

## Abstract (editor highlights)

Publisher’s note: Undertaking any experimental protocol requires adherence to local institutional guidelines for laboratory safety and ethics.

## Graphical abstract

**Figure:**
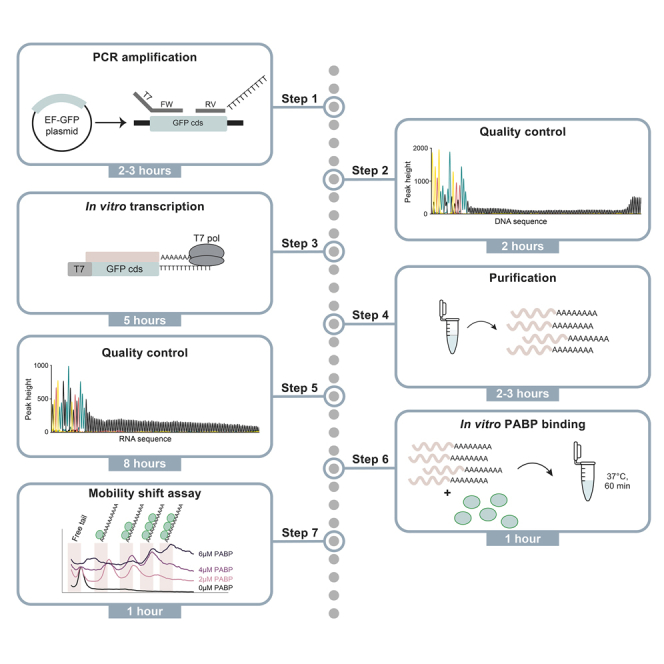

## Teaser

Quantifying the number of proteins that interact with mRNAs, in particular with poly(A) tails of mRNAs, is crucial for understanding gene regulation. Biochemical assays offer significant advantages for this purpose. Here, we present a protocol for synthesizing mRNAs with accurate, length-specific poly(A) tails through a PCR-based approach. We also describe steps for an *in vitro* (i.e., cell-free) approach for visualizing the sequential binding of PABPCs to these poly(A) tails. We detail quality control steps throughout the procedure.

## Before you begin

Poly(A)-Binding Proteins (PABPs) play a crucial role in regulating both degradation and translation of mRNAs by interacting with their poly(A)-tails.^2^^,^^3^^,^^4^^,^^5^ In differentiated cells, PABPs are typically present in excess compared to mRNAs.^3^^,^^6^^,^^7^ Under such non-limiting conditions, it is believed that the poly(A)-tail length and the number of PABP units bound to it correlate.^7^^,^^8^

Here we describe how to *in vitro* synthesize mRNAs with different and precise poly(A)-tail lengths, followed by the visualization of the sequential binding of PABPs using capillary electrophoresis. As purified proteins are notably expensive, our protocol has the advantage of minimizing the volumes used for the binding and the mobility shift assay.

***Note:*** While this protocol describes the synthesis of GFP-coding mRNAs, the reporter gene sequence can be customized depending on the application. The synthesized mRNAs can be further processed (i.e. 5′capped) and can be transfected into cells for follow-up analysis. While we have only focused on the *in vitro* binding of the mRNAs to PABPC1, the protocol should nevertheless work for other RNA Binding Proteins (RBPs) of interest, though this might require further optimization.

**CRITICAL:** All steps of the protocol should be performed with RNase-free reagents to prevent RNase contamination.

### Selection of DNA template of the reporter gene

**Timing: 1 h**

1. Choose the DNA template that includes the sequence of the reporter gene of your interest.

***Note:*** This can be a plasmid, PCR product, cDNA, genomic DNA, or synthetic DNA. In most cases, the templates do not contain the T7 promoter sequence required by the T7 polymerase to start transcription. This sequence can be added to the DNA template through a PCR reaction using specific primers, as described below. Here, we use the pEF-GFP^9^ plasmid as a starting point.

### Design of primers for the DNA template

**Timing: 1 h**

2. Design a forward primer considering the following:
  a. If your template does not contain the T7 promoter, add it by designing a forward primer containing its sequence.
    ***Note:***Table 1 reports an example of a basic forward primer sequence containing an upstream spacer, T7 promoter, downstream spacer, Kozak sequence, start codon, and a gene specific sequence of approximately 17–22 nt.
    **CRITICAL:** The inclusion of all these sequences is essential for enhanced ribosome binding, transcription initiation and translation initiation, in case the mRNA has to be expressed in cells.

**Table 1:** Basic primer sequence needed for the incorporation of the T7 promoter and other regulatory sequences in the template of interest

Segment	Feature	Sequence	Function
1	Upstream spacer	GC	Increase RNA polymerase binding
2	T7 promoter	TAATACGACTCACTATA**G**GG	Transcription initiation
3	Downstream spacer	ACAG	Increase ribosome binding
4	Kozak sequence	GCCACC	Translation initiation
5	Start codon	ATG	Translation initiation
6	Gene specific sequence	17-22 nt	Annealing of the template
  b. If your template already contains a T7 promoter and does not need the addition of any other regulatory sequence, design a 17–22 nt long forward primer specific for the beginning of your reporter gene.
3. The reverse primers should anneal to the terminal region of the 3′ UTR of your reporter gene and should contain a gene specific sequence of 17–22 nt, preceded by an oligo(dT) sequence of specified length (here we use 0, 5, 30, 50, 60, 100, 125 or 150 nt) at the 5′ end.

See Table 2 for the primer sequences used by us.

***Note:*** We found that for poly(A)-tails longer than 150 nt, the PCR and IVT products show too many bands, suggesting that unspecific products are being synthesized. Furthermore, increasing the sequence length before the T7 promoter could increase transcription efficiency. We therefore suggest not to make tails longer than 150 nt, or to implement additional optimization strategies, and to test different 5′ UTR sequences to determine the optimal yield.

**Table 2:** Primer sequences for the introduction of the T7 promoter and poly(A)-tails of different length

Primer	Sequence
Forward primer for the T7 promoter introduction	5′-GCTAATACGACTCACTATAGGGACAGGCCACCATGGTGAGCAAGGGCGAGGAGCTGT-3′
Reverse primer for the introduction of defined poly(A)-tail lengths	5′-T(5/30/50/60/100/125/150)CCCATATGTCCTTCCGAGTG-3′

### Design of primers and linkers for the DNA and mRNA quality control

**Timing: 1 h**

4. In the section “Quality control of the PCR products” we describe how to check the purity of the poly(A)-tailed PCR products by sequencing. For this, a gene specific forward primer of 17–22 nt is needed.

**Table 3:** Primers and linker sequences for the quality control of the PCR and mRNA products

Primer	Sequence
Forward primer for Sanger sequencing of PCR products	5′-AACGAGAAGCGCGATCACAT-3′
DNA linker for reverse transcription of mRNA products	5′-PO_4_-GGCACCATCAATCTTTTTT-NH_2_-3′
Reverse primer for reverse transcription and PCR of the linker-ligated mRNA products	5′-AAAAAAGATTGATGGTGCC-3′
Forward primer for PCR and Sanger sequencing of the linker-ligated mRNA products	5′-AACGAGAAGCGCGATCACATGGTCC-3′

***Note:*** The primer should bind 200–300 nt upstream to the poly(A)-tail, to ensure proper sequencing of the latter. See Table 3 for the primer sequence used by us.

5. To verify the purity of the poly(A)-tails on the mRNA products:
  a. Design a DNA linker that contains a PO_4_ modification at the 5′ end and a NH_2_ modification at the 3′ end and ligate it to the mRNA.
    ***Note:*** The 5′ phosphate is needed to facilitate the ligation to the RNA, while the 3' NH_2_ group prevents ligation of the linker to itself, and protects the linker from exonuclease activity, increasing the stability of the constructed molecule.
  b. The ligated mRNA product is reverse transcribed with a reverse primer containing a complementary sequence to the linker.
  c. The obtained cDNA is then amplified using a forward primer of 17–22 nt binding approximately 300 nt upstream of the poly(A)-tail, along with the identical reverse primer employed during the reverse transcription process.
  d. Finally, the amplified cDNA can be sequenced using the same forward primer utilized in the PCR step. See Table 3 for the sequences used by us.

### Preparation of buffers

**Timing: 2 h**

6. Before starting the protocol, make sure to have the buffers and solutions listed in the “materials and equipment” section ready.

## Key resources table

**Table:** 

REAGENT or RESOURCE	SOURCE	IDENTIFIER
**Recombinant DNA**		

pEF-GFP^9^	Addgene	Cat#11154

**Critical commercial assays**		

MEGAscript T7 transcription kit	Invitrogen	Cat#AMB13345
RNA high sensitivity kit	Invitrogen	Cat#Q32852
Agilent Nano 6000 RNA kit	Agilent Technologies	Cat#5067-1511
QIAquick PCR purification kit	QIAGEN	Cat#28104
QIAquick gel extraction kit	QIAGEN	Cat#28704

**Chemicals, peptides, and recombinant proteins**		

dNTP mix	Thermo Fisher Scientific	Cat#R0242
*Pfu* DNA polymerase buffer (10×)	In-house made, commercial alternative from Promega	Cat#M7741
*Pfu* DNA polymerase	In-house purified,^10^ commercial alternative from Promega	Cat#M7741
TURBO DNase	Thermo Fisher Scientific	Cat#AM2238
SuperScript III reverse transcriptase	Invitrogen	Cat#18080093
T4 RNA ligase	Thermo Fisher Scientific	Cat#EL0021
RiboLock RNase inhibitor (40 U/μL)	Thermo Fisher Scientific	Cat#EO0381
PABPC1	Biorbyt	Cat#ORB244425
TriTrack DNA loading dye (6×)	Thermo Fisher Scientific	Cat#R1161
GeneRuler DNA ladder mix	Thermo Fisher Scientific	Cat#SM0331
Ethidium bromide	Sigma-Aldrich	Cat#1239-45-8
Tris base	Sigma-Aldrich	Cat#T1503
EDTA	Sigma-Aldrich	Cat#E5134
DTT	Sigma-Aldrich	Cat#43819
PEG 8000	Sigma-Aldrich	Cat#202452
Boric acid	Sigma-Aldrich	Cat#B6768
MOPS	Sigma-Aldrich	Cat#M5162
Sodium acetate	Sigma-Aldrich	Cat#S2889
Formamide	Fisher Scientific	Cat#10592971
Formaldehyde solution 37%–41%	Fisher Scientific	Cat#10231622
MgCl_2_	Merck	Cat#05833
KCl	Merck	Cat#4938
NaOH	Merck	Cat#1.06498.1000
HCl	Sigma-Aldrich	Cat#30721
(NH_4_)_2_SO_4_	Sigma-Aldrich	Cat#A4418
BSA	Fisher Scientific	Cat#BP9702-100
Triton X-100	Sigma-Aldrich	Cat#T-9284
MgSO_4_	Merck	Cat#5886
Bromophenol blue dye	Merck	Cat#8122
Phenol:chloroform:isoamyl alcohol 49.5:49.5:1	Sigma-Aldrich	Cat#77619
Chloroform	LC	Cat#WH038
Isopropanol	Sigma-Aldrich	Cat#563935
Absolute ethanol	LC	Cat#WH254
LiCl	Sigma-Aldrich	Cat#L-8895

**Oligonucleotides**		

Primers and oligos for PCR, linker ligation and sequencing	Integrated DNA Technologies and Biolegio	N/A

**Software and algorithms**		

Python v3.8	Python Software Foundation^11^	https://www.python.org/
NumPy (Python package)	Harris et al.^12^	https://numpy.org/
Pandas	McKinney^13^	https://pandas.pydata.org/
Matplotlib (Python package)	Hunter^14^	https://matplotlib.org/
Seaborn	Waskom^15^	https://seaborn.pydata.org/

**Other**		

NanoDrop One	Thermo Fisher Scientific	Cat#ND-ONE-W
2100 Bioanalyzer instrument	Agilent Technologies	Cat#G2939BA
Qubit 4 fluorometer	Invitrogen	Cat#Q33238
0.5 mL Qubit assay tubes	Invitrogen	Cat#Q32856)

## Materials and equipment

### General stock solutions

#### 0.5 M EDTA, pH 8.0

Dissolve 18.61 g EDTA in 80 mL of RNase-free water. Dissolve the salt by adding sodium hydroxide (NaOH) pellets to the solution until the pH is 8.0. Top up the solution to 100 mL using RNase-free water and sterilize by autoclaving. Store at RT for a maximum of 1 year.

**CRITICAL:** EDTA can irritate skin, eyes, and the respiratory tract. Use protective equipment when handling it.

#### 0.1 M DTT

Dissolve 0.77 g DTT in 5 mL of RNase-free water. Bring the final volume to 10 mL with RNase-free water. Once in solution, store at −20°C for multiple years. Aliquot to avoid multiple freeze/thaw cycles.

#### 5 M LiCl

Dissolve 2.12 g LiCl in 5 mL of RNase-free water. Bring the final volume to 10 mL with RNase-free water. Store at RT for a maximum of 1 year.

**CRITICAL:** Lithium chloride is considered hazardous, as it may cause central nervous system effects, respiratory and digestive tract irritation and possible irritation or burns of the skin. Wear protective equipment when handling it.

#### 50% PEG 8000

Dissolve 5 g PEG 8000 in 5 mL of RNase-free water. Bring the final volume to 10 mL with RNase-free water. Store the solution at −20°C for a maximum of 1 year. Aliquot to avoid multiple freeze/thaw cycles.

#### 5 M (NH_4_)_2_SO_4_

Dissolve 66.07 g (NH_4_)_2_SO_4_ in 80 mL of RNase-free water. Bring the final volume to 100 mL with RNase-free water. Autoclave the solution and store at RT for a maximum of 1 year.

**CRITICAL:** (NH_4_)_2_SO_4_ can irritate the eyes, the skin and the respiratory tract. Wear protective equipment when handling it.

#### 1 M MgSO_4_

Dissolve 24.65 g MgSO_4_ in 80 mL of RNase-free water. Bring the final volume to 100 mL with RNase-free water. Autoclave the solution and store at RT for a maximum of 1 year.

#### 10 mg/mL BSA

Dissolve 100 mg BSA in 8 mL of RNase-free water. Bring the final volume to 10 mL with RNase-free water. Filter sterilize the final solution, and store it at 4°C.

#### 1 M Tris-HCl, pH 7.5 and pH 8.8

Dissolve 60.75 g Tris base in 400 mL of RNase-free water. Adjust pH to 7.5 or 8.8 with concentrated HCl. Bring final volume to 500 mL with RNase-free water. Autoclave the final solution and it store at RT for a maximum of 1 year.

**Caution:** Because HCl is a strong acid, do not inhale fumes and make sure to wear gloves when handling.

#### 1 M MgCl_2_

Dissolve 101.65 g MgCl_2_ in 400 mL of RNase-free water. Bring the final volume to 500 mL with RNase-free water. Autoclave the solution and store at RT for a maximum of 1 year.

#### 5 M KCl

Dissolve 186.38 g KCl in 400 mL of RNase-free water. Bring the final volume to 500 mL with RNase-free water. Autoclave the final solution and store it at RT for a maximum of 1 year.

### Buffers composition

**Table:** 10× *Pfu* DNA polymerase buffer

Reagent	Final concentration	Stock concentration	Add to 10 mL
Tris-HCl, pH 8.8	200 mM	1 M	2 mL
(NH_4_)_2_SO_4_	100 mM	5 M	200 μL
KCl	100 mM	5 M	200 μL
BSA	1 mg/mL	10 mg/mL	1 mL
Triton X-100	1%	100%	100 μL
MgSO_4_	20 mM	1 M	200 μL
RNase-free water	-	-	Up to 10 mL

Mix the solutions and store aliquots at −20°C. Avoid multiple freeze/thaw cycles.

**CRITICAL:** Triton X-100 can irritate the skin and eyes, organ toxicity and mutagenicity. Wear protective equipment when handling it.

**Table:** 10× TBE

Reagent	Final concentration	Stock concentration	Add to 1 L
Tris base	890 mM	-	107.8 g
Boric acid	890 mM	-	55 g
EDTA	25 mM	-	9.3 g
RNase-free water	-	-	Up to 1 L

Dissolve the reagents in 800 mL of RNase-free water. Adjust the final volume to 1 L with RNase-free water. Store at RT for a maximum of 1 year. If the buffer becomes cloudy or discolored, discontinue use and discard.

**CRITICAL:** Boric acid is considered hazardous and can cause reproductive toxicity. Avoid exposure and wear protective equipment.

**Table:** 10× MOPS

Reagent	Final concentration	Stock concentration	Add to 250 mL
MOPS	400 mM	-	20.91 g
Sodium acetate	100 mM	-	1.05 g
EDTA	10 mM	0.5 M	5 mL
RNase-free water	-	-	Up to 250 mL

Dissolve the reagents in 200 mL of RNase-free water and add the EDTA solution. Adjust the pH to 7.0 and the final volume to 250 mL with RNase-free water. Store the final solution 20°C–22°C protected from light for a maximum of 6 months.

**Table:** Formaldehyde Loading Dye

Reagent	Final concentration	Stock concentration	Add to 32 mL
Formamide (deionized)	62.5% (v/v)	100% (v/v)	20 mL
Formaldehyde	9.25% (v/v)	37% (v/v)	8 mL
10× MOPS buffer	1.25×	10×	4 mL

Mix the reagents and add bromophenol blue powder (a spatula tip is sufficient). Aliquot the solution and store at −20°C for a maximum of 1 year. Try to minimize freeze/thaw cycles.

**CRITICAL:** Formaldehyde and formamide are toxic when inhaled, ingested, or absorbed through the skin. Work in a fume hood and wear protective goggles. Bromophenol blue is recognized as environmentally hazardous. Handle it with caution, ensuring proper storage, ventilation, and personal protective equipment to minimize environmental impact and prevent harm.

**Table:** 5× Binding Buffer

Reagent	Final concentration	Stock concentration	Add to 15 mL
Tris-HCl, pH 7.5	100 mM	1 M	1.5 mL
MgCl_2_	100 mM	1 M	1.5 mL
KCl	1 M	5 M	3 mL
RNase-free water	**-**	**-**	9 mL

Mix the solutions and store the buffer at RT for maximum 1 year.

### Gel electrophoresis

**Table:** 1.2% non-denaturing agarose gel

Reagent	Final concentration	Amount
Agarose	1.2%	0.6 g
1× TBE buffer	1×	50 mL
Ethidium Bromide	0.5 μg/mL	Variable depending on stock
**Total**	**N/A**	**50 mL**

**CRITICAL:** EtBr is a potent mutagen and therefore needs to be handled with care, wearing protective clothing and gloves.

***Alternatives:*** Safer and non-mutagenic alternatives to Ethidium Bromide are SYBR Green, GelRed, GelGreen and EvaGreen. It is also possible to analyze the PCR products by capillary electrophoresis using Agilent DNA Kits (Bioanalyzer).

**Table:** 1.2% denaturing agarose gel

Reagent	Final concentration	Amount
Agarose	1.2%	0.6 g
Water	-	36 mL
10× MOPS	1×	5 mL
37% formaldehyde	6.66%	9 mL
**Total**	**N/A**	**50 mL**

## Step-by-step method details

### Addition of T7 promoter and poly(A)-tail sequence to the DNA template

**Timing: 2–3 h**

The pEF-GFP plasmid is amplified with a forward primer that allows the incorporation of the T7 promoter sequence and a reverse primer with variable poly(dT) sequences (see Table 2 for the sequences used by us). This allows the incorporation of poly(A)-tails of defined lengths to the DNA template.

1. Thaw all reagents at RT and keep on ice.
2. Add to a 0.2 mL tube the PCR components, as specified in Table 4. Set up the reaction on ice.

**Table 4:** Reagents for a single 100 μL PCR reaction for the introduction of the poly(A)-tails

Component	Final concentration	Stock concentration	Amount
RNase-free water	-	-	Up to 100 μL
*Pfu* DNA polymerase buffer	1×	10×	10 μL
dNTP mix	0.2 mM	2 mM	10 μL
F primer 10 μM	1 μM	10 μM	10 μL
R primer 10 μM	1 μM	10 μM	10 μL
DNA template	150 pM	Variable	Variable
*Pfu* DNA polymerase	-	-	2.5 units
**Tot. Volume**			**100 μL**

**CRITICAL:** For in-house purified DNA polymerases it is important to determine the enzyme activity prior use (defined as the amount of enzyme required to incorporate 1 nmol of nucleotides per minute). We have determined the activity of our enzyme by measuring the incorporation of fluorescently labelled nucleotides, but this can also be calculated through spectrophotometric assays, by measuring changes in absorbance due to the incorporation of nucleotides or the production of pyrophosphate.

3. Incubate the reaction in a thermocycler with pre-heated lid (95°C) following the conditions reported in Table 5.

**Table 5:** PCR program used for the synthesis of tailed PCR products

Steps	Temperature	Time	Cycles
Initial Denaturation	95°C	2 min	1
Denaturation	95°C	30 s	25 cycles
Annealing	57°C	30 s	
Extension	72°C	1 min	
Final extension	72°C	10 min	1
Hold	15°C	Infinite

***Note:*** 30 s of annealing time is a consensus time. Longer or shorter times can be applied depending on the behavior of the primers or PCR yield.

***Note:*** Cycle elongation time can vary depending on the length of the DNA that is amplified. Generally, use 1 min per 1000 bp for the *Pfu* DNA polymerase.

***Note:*** We used 5°C per second as ramp time between the steps, but also lower speeds can be used.

***Note:*** Over-cycling may result in undesired side reactions, especially impacting the accuracy of the poly(A)-tail length. In fact, the misalignment of the tailed reverse primer to the newly synthesized tailed products can lead to the synthesis of PCR products with unspecific poly(A)-tail length, which will get further amplified in the following cycles.

***Note:*** The amount of template reported in the table is optimized for the pEF-GFP plasmid. This can vary depending on the DNA template used:

 5–100 ng Plasmid DNA

 5–50 ng PCR product

 10–1000 ng Genomic DNA.

4. Purify the PCR products with the QIAGEN PCR purification kit, following the manufacturer’s instructions.
  ***Note:*** Any alternative purification method is good as long as the final PCR products are free from contaminants that could potentially disrupt following reactions.
  a. Perform all centrifugation steps at 17900 g using a microcentrifuge at 20°C–22°C.
5. Quantify 1 μL of purified PCR product with a NanoDrop spectrophotometer:
  a. Select the protocol for dsDNA.
  b. Blank using the elution buffer used in step 4h.
  c. Add 1 μL of purified PCR product and measure the concentration.

**CRITICAL:** The A260/A280 and A260/A230 ratios are important to assess the purity of the samples. An A260/A280 ratio of approximately 1.8 indicates pure DNA. Lower values may imply contamination with proteins, phenol or other substances absorbing around 280 nm. The expected A260/230 ratio falls within the range 2.0–2.2. If ratios are below the expected ones, DNA samples should be re-purified by phenol/chloroform extraction in case of protein contaminants, or ethanol precipitation in case of the presence of small organic molecules. In case of RNA contamination, which contributes to the absorbance at 260 nm, samples should be treated with RNase.

**Pause point:** The resulting PCR products can be stored for multiple months at −20°C.

### Quality control of the tailed PCR products

**Timing: 2 h**

Before proceeding with IVT, it is recommended to check the quality of the tailed PCR products utilizing gel electrophoresis and DNA sequencing. Gel electrophoresis can highlight the presence of unspecific products and is crucial for verifying the expected size of the inserted poly(A)-tails. On the other hand, DNA sequencing ensures that the poly(A)-tails do not contain non-A nucleotides, which might be introduced by the DNA polymerase with different efficiencies depending on the poly(A)-tail length.

**Figure 1:**
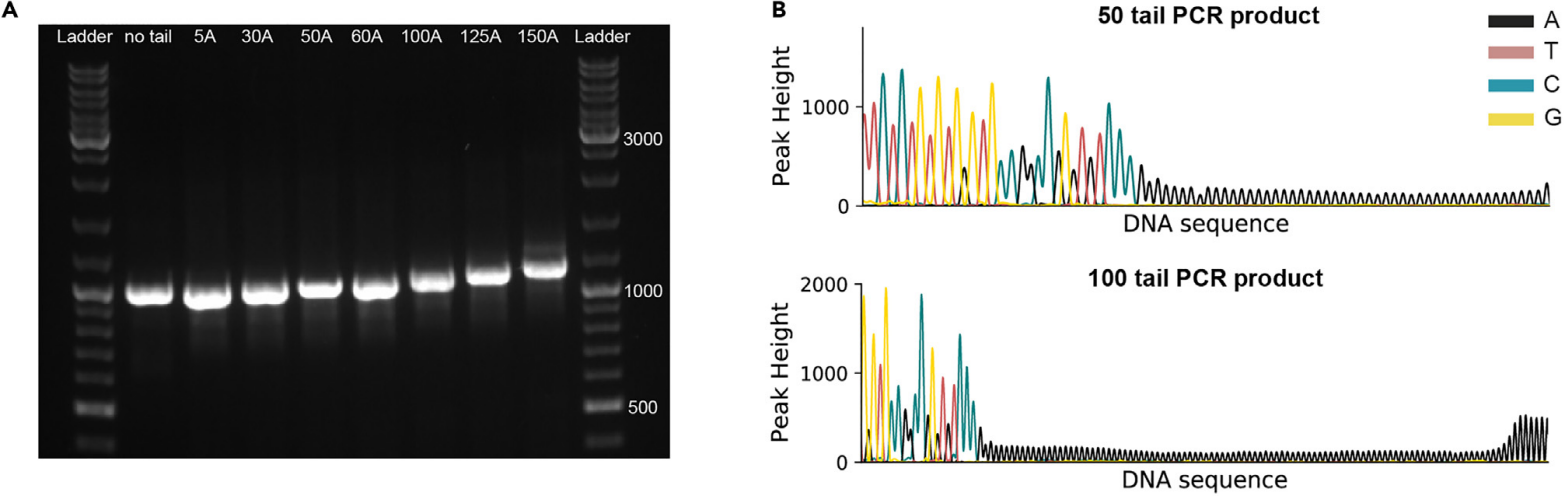
Quality control of the poly(A)-tail PCR products (A) Non-denaturing agarose gel showing the PCR products with increasing poly(A)-tail length. (B) Sanger sequencing of the PCR product containing a 50 or 100 nt long poly(A)-tail.
7. Sequence the PCR products using a forward primer that binds at the 3′UTR and double-check that the poly(A)-tail does not contain non-A nucleotides (see Figure 1B in expected outcomes). The primer sequence used in this protocol is reported in Table 3.

***Note:*** We chose Sanger sequencing from BaseClear for our PCR products, but any type of DNA sequencing that can accurately read the poly(A)-tail will work.

6. Make a 1.2% non-denaturing agarose gel:
  ***Note:*** The percentage of the agarose gel depends on the size of the expected PCR products.
  a. Mix 250 ng of PCR products with 1 μL of TriTrack DNA Loading Dye (6×) and add water up to 6 μL.
  b. Mix 3 μL of GeneRuler DNA Ladder Mix with 3 μL of water in a clean tube.
  c. Pipette the Ladder and the PCR products into the wells of the solidified gel.
  d. Run the gel in TBE 1× buffer using a voltage between 90–110 V until the DNA bands have migrated to the desired position.
  e. Remove the gel form the tray and inspect it on a UV transilluminator.
  f. The bands should be of the expected size (in this case 976 nt + the length of the added poly(A)-tail) and the size should increase with the poly(A)-tail length (see Figure 1A in expected outcomes).

**CRITICAL:** The presence of non-A nucleotides in the PCR products will be propagated in the mRNA molecules. These nucleotides can alter the interaction with the PABPs^16^ but are also known to impact translation and degradation.^17^^,^^18^^,^^19^^,^^20^ If non-A peaks are observed, the decision of continuing with IVT of the PCR products depends on the following applications. However, if the molecules are used for kinetic and binding analysis, we suggest discarding molecules that show clear peaks associated to non-A nucleotides.

### *In vitro* transcription of poly(A)-tailed GFP coding mRNAs

**Timing: 9–10 h**

The tailed PCR products are *in vitro* transcribed to generate mRNAs with defined poly(A)-tail lengths.

***Note:*** The protocol describes the mRNA synthesis using the MEGAscript T7 Transcription Kit (Invitrogen), but any other T7-based transcription kit can also be used. If the T7 wild-type polymerase is exchanged with mutant polymerases, we recommend carefully checking the quality of the final mRNA products.

8. Thaw and vortex the reagents:
  a. Keep the RNA Polymerase Enzyme Mix on ice.
  b. Keep the 10× Reaction Buffer at 20°C–22°C.

**CRITICAL:** The reaction must be set up at 20°C–22°C because the spermidine in the 10× Reaction Buffer can cause the template DNA to coprecipitate if assembled on ice. Ensure you add the 10× Reaction Buffer after the water and ribonucleotides.

9. Mix the reagents reported in Table 6.

**Table 6:** Reagents for a single 20 μL IVT reaction

Component	Final concentration	Stock concentration	Amount
RNase-free water	-	-	Up to 20 μL
ATP solution	7.5 mM	75 mM	2 μL
CTP solution	7.5 mM	75 mM	2 μL
GTP solution	7.5 mM	75 mM	2 μL
UTP solution	7.5 mM	75 mM	2 μL
Reaction Buffer	1×	10×	2 μL
PCR product	15 nM	Variable	Variable
Enzyme Mix	1×	10×	2 μL
**Tot. Volume**			**20 μL**

***Note:*** Volumes are for a 20 μL reaction, but they can be scaled up if needed.

10. Incubate the reactions at 37°C for 4 h.

***Note:*** The incubation time depends on the size and transcriptional efficiency of the template, and therefore could vary. For transcripts shorter than 500 nucleotides, extending the incubation time to approximately 16 h can be beneficial. This is because a greater number of transcription initiation events are necessary to produce a specific quantity of RNA compared to longer transcripts.

11. Add 1 μL of TURBO DNase, mix well and incubate at 37°C for 15 min.
12. Purify the mRNAs with following the desired protocol.
  ***Note:*** Here we present two types of purification methods, you can choose the most suitable one depending on your application.
  a. Phenol:Chloroform extraction followed by and isopropanol precipitation.
    ***Note:*** This method is the most thorough for purifying transcripts, effectively eliminating all enzymes and most free nucleotides.
    i. Bring the reaction volume to 500 μL by adding RNase-free water. Mix well.
    ii. Add a mixture consisting of an equal volume of Phenol:Chloroform:Isoamyl Alcohol.
      **CRITICAL:** Phenol:Chloroform:Isoamyl Alcohol is toxic if inhaled, and can cause eye damage and severe skin burns. Prolonged exposure can damage organs. When handling, work in a fume hood and wear protective goggles.
    iii. Vortex samples for 10 s.
    iv. Incubate samples for 2–3 min at 20°C–22°C.
    v. Centrifuge for 15 min at 4°C, 12000 g.
    vi. Transfer the aqueous phase into a clean tube.
    vii. Add 1 volume of chloroform (1:1 ratio aqueous phase layer:chloroform).
      **CRITICAL:** Chloroform can be toxic if inhaled. Long exposure may also cause cancer. When handling, work in a fume hood and wear protective goggles.
    viii. Vortex samples for 10 s.
    ix. Incubate samples for 2–3 min at 20°C–22°C.
    x. Centrifuge for 15 min at 4°C, 12000 g.
    xi. Transfer again the aqueous phase into a clean tube.
    xii. Add 1 volume of isopropanol and mix it by reversing 5–6 times.
    xiii. Incubate at −20°C for at least 30 min.
    xiv. Centrifuge for 15 min at 4°C, 12000 g.
    xv. Dispose of the supernatant.
    xvi. Wash the pellet with 500 μL of ice cold 70% ethanol.
      **CRITICAL:** You should only rinse the pellet, not resuspend it.
    xvii. Centrifuge for 5 min at 4°C, 7500 *g*.
    xviii. Dispose of the supernatant.
    xix. Allow the pellet to air dry.
    xx. Reconstitute the pellet in RNase-free water by gently pipetting up and down.
  b. Lithium chloride precipitation.
    ***Note:*** While this method efficiently eliminates unincorporated nucleotides and the majority of proteins, it is not ideal for precipitating RNAs smaller than 300 nucleotides. To ensure effective precipitation, the RNA concentration should be > 0.1 μg/μL.
    i. Add 30 μL of RNase-free water and 30 μL of 5 M LiCl (final concentration 2.5 M) and mix thoroughly.
    ii. Incubate samples at −20°C for at least 30 min.
    iii. Centrifuge samples at 4°C for 15 min and top speed.
    iv. Dispose of the supernatant.
    v. Wash the pellet with 1 mL of ice cold 70% ethanol.
      **CRITICAL:** You should only rinse the pellet, not resuspend it.
    vi. Centrifuge for 15 min at 4°C at top speed.
    vii. Remove the supernatant.
    viii. Allow the pellet to air dry.
    ix. Reconstitute the pellet in RNase-free water by gently pipetting up and down.
      ***Note:*** If the pellet is not properly dissolving in water, you could try to heat up the samples to 37°C.
      **CRITICAL:** Try to minimize the volume used for resuspension, in order to have highly concentrated products.
13. Quantify the purified RNA using a Qubit RNA kit (High Sensitivity or Broad Range), following manufacturer’s instructions.

***Note:*** If the IVT reaction yield is high, you most likely will have to dilute your samples before quantification as their concentration will be out of range and the Qubit measurement will not be possible. We recommend performing 3 dilution replicates per sample and finally calculate their average concentration to have a reliable and accurate quantification.

**Pause point:** The purified IVT products can be stored at −20°C or −80°C for multiple months. Avoid freeze/thawing cycles. We suggest making aliquots of your samples to minimize these.

### Quality control of the IVT products

**Timing: 8–10 h**

It is recommended to check the quality of the IVT products by gel or capillary electrophoresis and sequencing. The electrophoresis analysis can highlight the presence of unspecific products and is crucial for verifying the expected size of the mRNA and of the inserted poly(A)-tails. On the other hand, sequencing ensures that the poly(A)-tails do not contain non-A nucleotides, which might be introduced by the T7 RNA polymerase with different efficiencies depending on the poly(A)-tail length.

14. Here we explain how to inspect the IVT products size.

***Note:*** We recommend using capillary electrophoresis with the Bioanalyzer, Agilent RNA 6000 Nano kit, as it has a higher sensitivity compared to classic gel electrophoresis and can therefore more efficiently show the presence of unspecific products. However, we also describe the protocol for a denaturing agarose gel, which is a cheap and fast way to visualize long RNA transcripts, and separates more efficiently RNA molecules > 1000 nt, although with lower resolution than polyacrylamide gel electrophoresis (PAGE).

15. Run samples with Bioanalyzer, Agilent RNA 6000 Nano kit:
  a. Load the samples on the chip following manufacturer’s instructions.
  b. In the Instrument context, select the mRNA Nano assay from the Assay menu (Electrophoresis → RNA → mRNA Nano assay).
    ***Note:*** Once you start the chip run, the incoming raw signals will be displayed in the Instrument context.
  c. To check the results of your run, select the Gel or Electropherogram tab in the Data context.
    ***Note:*** A successful run should show clear distinct peaks for the ladder.
  d. To examine the outcomes of a specific sample, choose the sample name in the tree view.
    ***Note:*** The electropherogram of a clean *in vitro* synthesized mRNA should show a single peak corresponding to the mRNA (see Figure 2A in expected outcomes).

**Figure 2:**
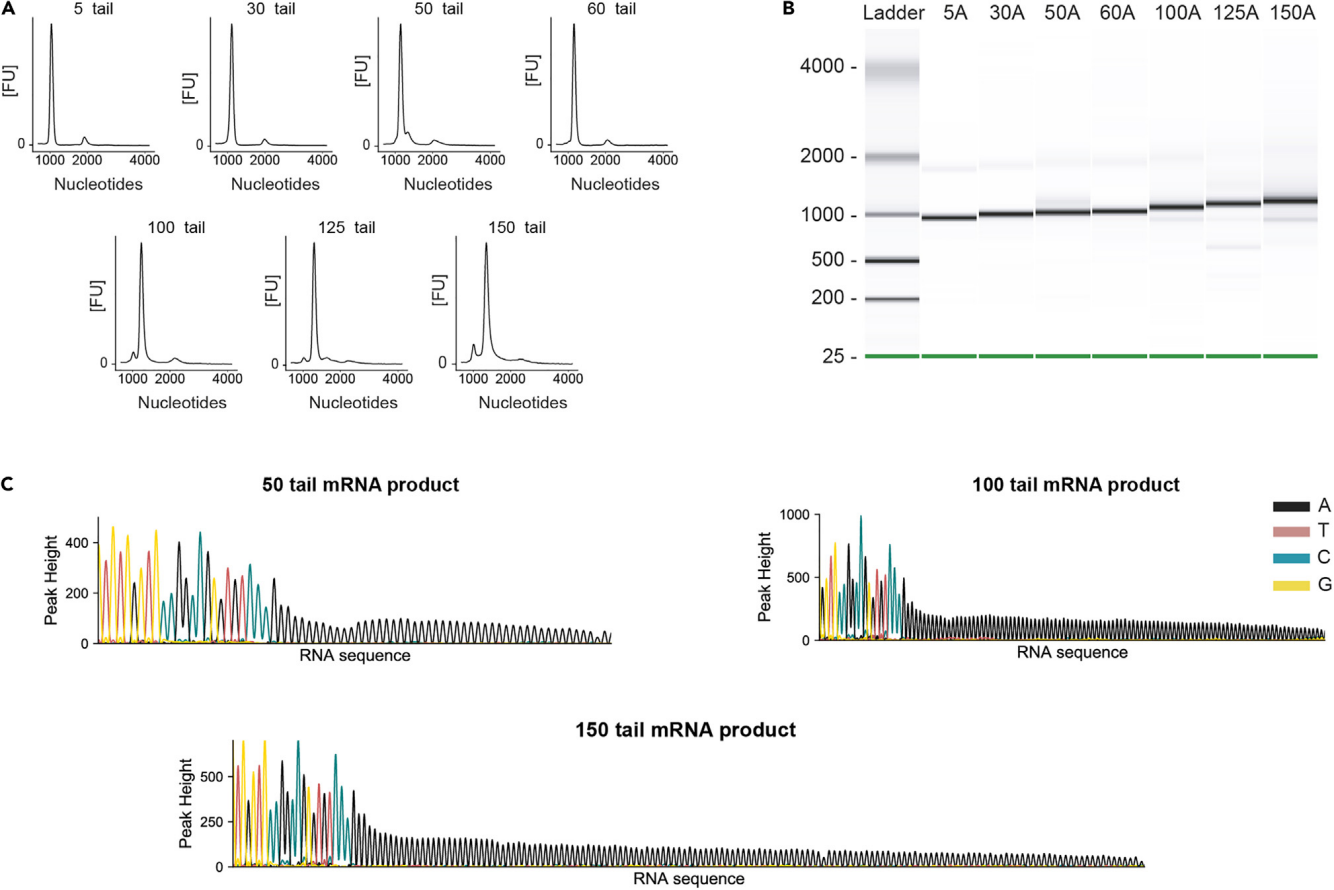
Quality control of the poly(A)-tailed mRNA products (A) Peaks obtained by capillary electrophoresis for mRNAs with increasing poly(A)-tail length. (B) Gel-like image obtained by capillary electrophoresis showing the size of the *in vitro* transcribed poly(A)-tailed mRNAs. (C) Sequence of 50, 100 and 150 nt long poly(A)-tailed mRNAs obtained from Sanger sequencing.
  e. To visualize the results in a gel-like form, click on the Gel tab.
    ***Note:*** Clean RNA should behave similarly to the PCR products, where longer tails progressively show increasing size (see Figure 2B in expected outcomes).
    ***Note:*** By clicking on Gel → Show sizes, seconds will be displayed as nucleotides.
  f. Enhance the contrast of single lanes in the Agilent Bioanalyzer 2100 Expert Software to increase the signal.
16. Make a denaturing agarose gel 1.2%:
  **CRITICAL:** Formaldehyde is toxic when inhaled, ingested, or absorbed through the skin. It is therefore essential to work in a fume hood and wear protective goggles.
  a. In clean tubes, mix 300 ng of IVT products or RNA ladder with 7.5 μL of Formaldehyde Loading Dye.
  b. Add Ethidium Bromide (EtBr) to a final concentration of 0.5 μg/mL directly in the sample tubes.
  c. Incubate the samples at 75°C for 10 min.
  d. Pipette the Ladder and the IVT products into the wells of the solidified gel.
  e. Run the gel in 1× MOPS using a voltage of 90–110 V until the bands have migrated to the desired position.
  f. Remove the gel form the tray and inspect it on a UV transilluminator.
  g. The bands should be of the expected size and should increase with increased poly(A)-tail length.
17. If the mRNAs show the expected size and no unspecific products, you can proceed with sequencing to inspect the quality of the poly(A)-tail.
  ***Note:*** The sequencing is not performed directly on the RNA, but on reverse transcribed and PCR amplified product.
  a. Ligate the DNA oligonucleotide linker modified with 5′PO_4_ and 3′NH_2_ to the 3′ end of the mRNAs with different poly(A)-tail lengths.
  b. Assemble a 25 μL ligation reaction as indicated in Table 7.

**Table 7:** Reagents for a single 25 μL ligation reaction

Component	Final concentration	Stock concentration	Amount
RNase-free water	-	-	7.5 μL
T4 RNA ligase buffer	1×	10×	2.5 μL
ATP	1 mM	10 mM	2.5 μL
PEG 8000	12.5%	50%	6.25 μL
RNA	-	-	50 ng
Linker	1–2 μM	50 μM	0.5–1 μL
T4 RNA ligase	-	-	10 units
Ribolock	-	-	40 units
**Tot. Volume**			**25 μL**
  c. Place the reaction mixture in a thermomixer and incubate it for 3 h at 25°C while shaking at 500 rpm.
  d. Quench the ligation reaction by adding 0.5 μL EDTA (0.5 M, pH 8.3).
  e. Aliquot 2.5–5 μL of ligated RNA products for reverse transcription.
  f. Reverse transcribe the ligated RNA products using a DNA primer complementary to the linker (see Table 3 for the sequence used by us).
  g. Assemble the reverse transcription mixture in a 20 μL reaction as indicated in Table 8.
    ***Note:*** The amount of linker-ligated RNA can be varied to improve the product quality and/or yield if desired. Generally, within these boundaries the result should be satisfactory.

**Table 8:** Reagents for a single reverse transcription reaction of the linker-ligated products

Component	Final concentration	Stock concentration	Amount
RNase-free water	-	-	6–8.5 μL
First strand buffer	1×	5×	4 μL
DTT	5 mM	0.1 M	1 μL
dNTP mix	0.5 mM	10 mM	1 μL
Linker-ligated RNA	-	-	2.5–5 μL
RT primer	0.5 μM	10 μM	1 μL
**Tot. Volume**			**18 μL**
  h. Heat the mixture at 70°C for 2 min and let it cool down to 20°C–22°C.
  i. Briefly centrifuge contents at 10000 × *g* in a table top centrifuge.
  j. Add 1 μL of Ribolock (40 U/μL) and 1 μL of Superscript III (200 U/μL) and mix.
  k. Place the reactions at 48°C for 40 min.
  l. Halt the reaction and aliquot 2.5 μL of reverse transcription mixture for PCR amplification.
  m. Assemble three PCR mixtures of 20 μL each by adding the reagents as indicated in Table 9 (see Table 3 for the primer sequences used by us).

**Table 9:** Reagents for a 20 μL PCR reaction for the amplification of the reverse transcribed cDNA products

Component	Final concentration	Stock concentration	Amount
RNase-free water	-	-	Up to 20 μL
*Pfu* DNA polymerase buffer	1×	10×	2 μL
dNTP mix	0.2 mM	2 mM	0.5 μL
F primer	1 μM	10 μM	2 μL
R primer	1 μM	10 μM	2 μL
cDNA template	-	-	2.5 μL
*Pfu* DNA polymerase	-	-	0.5 units
**Tot. Volume**			**20 μL**
  n. Incubate the reaction in a thermocycler following the conditions reported in Table 10.

**Table 10:** PCR program used for the amplification of the cDNA products

Steps	Temperature	Time	Cycles
Initial Denaturation	95°C	2 min	1
Denaturation	95°C	30 s	15-17 cycles
Annealing	49°C	30 s	
Extension	72°C	1 min	
Final extension	72°C	10 min	1
Hold	15°C	Infinite	

**Note:** The number of cycles can be varied to improve the product quality and/or yield if desired. Generally, within these boundaries the result should be satisfactory.
  o. Mix the sample with 4 μL of TriTrack DNA Loading Dye (6×) and load it on a 1% agarose gel, as explained in step 6.
    ***Note:*** The fragment of interest should be sharp at this point with minimal smearing or other fragments surrounding it.
  p. Excise the amplified DNA products from the gel under UV light and purify them using a QIAquick gel extraction kit:
    i. Cut the DNA fragment from the gel with a scalpel.
    ii. Purify the DNA fragment in a QIAquick Spin Column following the manufacturer’s instructions.
  q. Sequence the PCR products using a forward primer that binds 200–300 nt upstream to the poly(A)-tail and check that the poly(A)-tail does not contain non-A nucleotides (see Figure 2C in expected outcomes). See Table 3 for the primer sequence used by us.
    ***Note:*** We chose Sanger sequencing from BaseClear for our PCR products, but any type of DNA sequencing that can accurately read the poly(A)-tail will work.

### *In vitro* PABPCs binding assay and mobility shift assay

**Timing: 2 h**

After having synthesized the desired poly(A)-tailed mRNA transcripts, you can proceed with the *in vitro* PABPCs binding and visualization through shift mobility assay.

***Note:*** As purified proteins are notably expensive, the protocol presented here minimizes the reaction volumes (i.e., only 5 μL), allowing the visualization of very low amounts of RNA with bound PABPCs, which would not be visible on an agarose gel.

18. Proceed by assembling the reactions for the *in vitro* binding.
  a. Mix the components indicated in Table 11 in a 0.5 mL PCR tube (assemble on ice):
    ***Note:*** We recommend quantifying the PABP concentration before usage, as this might drastically differ from batch to batch. Furthermore, we recommend using 0.5 mL PCR tubes as they can efficiently collect the small volume used in the reaction at their bottom.

**Table 11:** Reagents for a 5 μL PABP binding reaction to poly(A)-tailed mRNAs

Component	Amount
RNase-free water	Up to 5 μL
5× Binding Buffer	1 μL
PABPC1	0/2/4/6 μM
mRNA	0.5 μM
**Tot. Volume**	**5 μL**
  b. Mix well and spin down.
  c. Incubate the reactions at 37°C for 60 min in a PCR machine with a heated lid.
  d. In the meantime, prepare the Bioanalyzer, Agilent RNA 6000 Nano Chip as described in step 14a. Do not heat denature the samples.
  e. After incubation, load immediately 1 μL of the binding reactions in each well of the Bioanalyzer chip.
    **CRITICAL:** Do not put the reactions on ice, but transfer them directly from the thermocycler to the chip. Run the Bioanalyzer chip immediately after loading.
  f. Place the chip in the Bioanalyzer.
  g. Select the mRNA Nano assay from the Assay menu (Electrophoresis → RNA → mRNA Nano assay).

### Data analysis and processing

**Timing: 30 min–1 h**

19. After running, visualize the data as explained in step 14a.
20. Export raw data to further process and normalize. To export the csv files:
  a. Click on File → Export. The “Electrophoresis Export Option” window will open.
  b. Check the “Result Tables” to export a csv file containing all result table values (make sure to include the ladder).
  c. Check the “Sample Data” tab to export one file per sample.
  d. Check the “Gel Image” tab to export the gel like image (see Figure 3A in expected outcomes for some examples).

**Figure 3:**
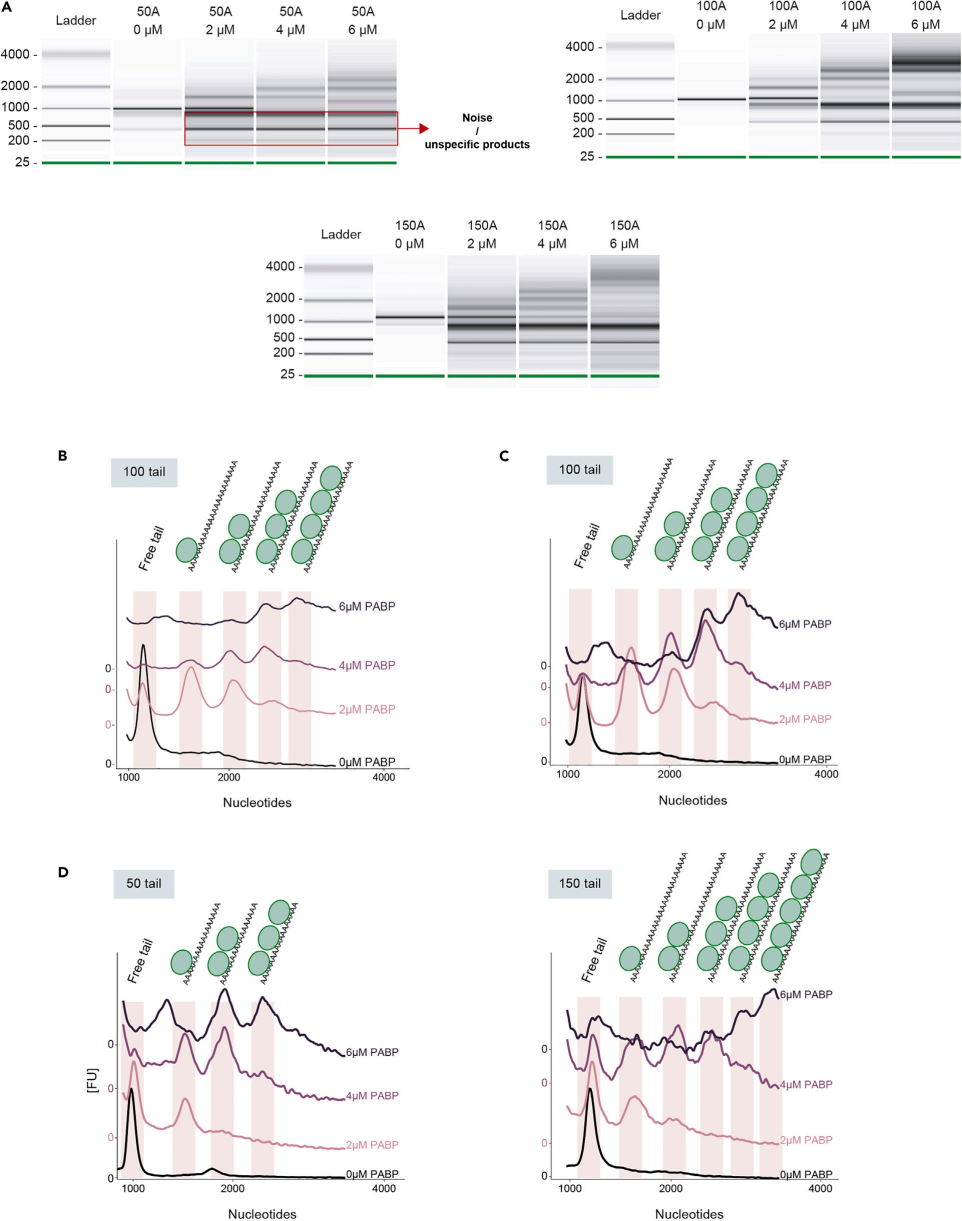
Mobility shift assay of the sequential PABP binding to the poly(A)-tail. (A) Gel-like images obtained by capillary electrophoresis for the *in vitro* binding of PABPC1 to mRNAs with 50, 100 and 150 nt long poly(A)-tails (50A 100A 150A). These tails respectively bind 3, 4 and 5 PABPC1 units. Samples for each poly(A)-tail length are from the same run. Concentrations shown are final PABPC1 concentrations. The contrast of single lanes is enhanced in the Agilent Bioanalyzer 2100 Expert Software to increase the signal. The red box highlights bands associated to noise or unspecific products, which become more evident as the signal-to-noise ratio decreases, due to the dilution of the mRNA throughout the multiple bands. (B) Electrophoresis peaks corresponding to single PABP units binding to 100 nt long poly(A)-tail, normalized to the marker of each lane. Zero values on the y axis correspond to the minimum value of each assay (0 μM, 2 μM, 4 μM and 6 μM PABP). (C) Electrophoresis peaks corresponding to single PABP units binding to 100 nt long poly(A)-tail, normalized to the highest peak value of each sample. Zero values on the y axis correspond to the minimum value of each assay (0 μM, 2 μM, 4 μM and 6 μM PABP). (D) Electrophoresis peaks corresponding to single PABP units binding to 50 and 150 nt long poly(A)-tail, normalized as in (C).
21. The raw data contained in the csv files exported from the Agilent Bioanalyzer 2100 Expert Software can be processed and plotted in Python. Here we show how to normalize the Bioanalyzer peaks in two ways:
  a. To the marker peak.
  b. To the highest peak of each sample.
22. To do this, you will need the following files:
  a. One csv file containing the Ladder information.
    ***Note:*** When you export the “Result Table” csv file, make sure to check the tab “Include Ladder”. You can then just copy the Ladder peak table in a new csv file (for an example see the provided file 'Ladder_peaks.csv').
  b. A separate csv file for each sample:
    i. export the Aligned Sample Data tables from the Agilent Bioanalyzer 2100 Expert Software (see the csv files “Sample_0.csv”, “Sample_2.csv”, “Sample_4.csv” and “Sample_6.csv” as an example).
      ***Note:*** Here we use Jupyter Notebook with Python v3.8 as computing platform, but any other Python-based platform will work.
23. Install the required packages using “pip”:

pip install pandas numpy matplotlib seaborn

24. Import the required packages and the color palette of your choice:

import os

import pandas as pd

import numpy as np

import matplotlib.pyplot as plt

import seaborn as sns

%matplotlib inline

color=sns.color_palette("ch:s=-.0,r=.4")

25. Define the general function needed to normalize the data to a reference peak (which can be the marker or the highest peak of the sample):

def Normalize_to_ref(data, ref):

 """

 Normalizes data with respect to a reference using scaling.

 Parameters:

 data (array-like): Input data to be normalized.

 ref (array-like): Reference data.

 Returns:

 array-like: Normalized data with respect to the reference.

 """

 return (data) / (ref)

26. Define the csv files to import and the data directory.

***Note:*** The number used in the dictionary refers to the concentration of PABP used in the experiment. This can be customized, as long as the number in the dictionary corresponds to the one in the csv file name.

file_dir = {

 0 : "0μM PABP",

 2 : "2μM PABP",

 4 : "4μM PABP",

 6 : "6μM PABP",

}

DATA_DIR = 'insert_your_path/'

27. Define the plot settings:

fig, ax = plt.subplots(1, 1, sharex=True, sharey=True)

plt.rcParams['text.usetex'] = True

plt.style.use('default')

plt.gcf().set_size_inches(7, 5)

palette = {file_nr: color[i] for i, file_nr in enumerate(file_dir.keys())}

28. To normalize the data to the marker peak:
  a. Normalize the control sample first (the one with 0 μM PABP) and plot it:
    """
    Specify the control sample (i.e. the one with 0 μM PABP)
    """
    control = 0
    No_PABP = pd.read_csv(DATA_DIR+'Sample_'+str(control)+'.csv',
           skiprows=16,
           skipfooter=1,
           engine='python')
    """
    Specify in which range of seconds the marker peak occurs and get the max value of this peak (it should be approximately at 19 seconds)
    """
    No_PABP_norm = No_PABP.query('18.8 <= Time <= 19.2')
    ref_peak = No_PABP_norm.Value.max()
    """
    Specify in which range of seconds the sample peaks occur and normalize them to the max value of the marker peak, then plot
    """
    No_PABP = No_PABP.query('31.5 <= Time <= 42')
    No_PABP["Value_norm"] = Normalize_to_ref(No_PABP.Value, ref_peak)
    ax.plot(No_PABP.Time, No_PABP.Value_norm, color='k', lw=2, alpha=1)'
  b. Do the same with each sample containing PABP and plot with vertical shift.
    """
    Define the vertical shift
    """
    move = 1
    for file_nr, name in file_dir.items():
     """
     Skip control sample, since that is already plotted
     """
     if file_nr == control:
      continue
     electrogram =
     pd.read_csv(DATA_DIR+'Sample_'+str(file_nr)+'.csv',
              skiprows=16,
              skipfooter=1,
              engine='python')
    """
    Find the marker peak of each sample (it should be approximately at 19 seconds)
    """
    electrogram_norm = electrogram.query('18.8 <= Time <= 19.2')
    ref_peak = electrogram_norm.Value.max()
    """
    Specify in which range of seconds the sample peaks occur and
    normalize them to the max value of the marker peak
    """
    electrogram = electrogram.query('31.5 <= Time <= 42')
    electrogram["Value_norm"] =
    Normalize_to_ref(electrogram.Value, ref_peak)
    ax.plot(electrogram.Time, electrogram.Value_norm + move,
      color=palette[file_nr], lw=2)
    move += 1
  c. Annotate the plot.
    for line, name in zip(ax.lines, file_dir.values()):
     y = line.get_ydata()[-1]
     x = line.get_xdata()[-1] + 0.2
     if not np.isfinite(y):
      y = next(reversed(line.get_ydata()[∼line.get_ydata().mask]),float("nan"))
     if not np.isfinite(y) or not np.isfinite(x):
      continue
     text = ax.annotate(name,
            xy=(x, y),
            xytext=(0, 0),
            color=line.get_color(),
            xycoords=(ax.get_xaxis_transform(),
                ax.get_yaxis_transform()),
            textcoords="offset points", fontsize=12)
     text_width = (text.get_window_extent(
         fig.canvas.get_renderer()).transformed(
         ax.transData.inverted()).width)
     if np.isfinite(text_width):
      ax.set_xlim(ax.get_xlim()[0], text.xy[0] + text_width ∗ 1.05)
  d. Import the csv file containing the information about the ladder peaks.
    ***Note:*** Here we select the peaks relative to the 1000, 2000 and 4000 nt bands, and we use this information to add the ticks on the x axis.
    **CRITICAL:** Make sure to manually check the cropped/assigned markers. Sometimes the Bioanalyzer software assigns multiple peaks which are not annotated as marker peaks. So the range of peaks [3:−1] could differ from run to run in the ladder csv file.
    peak_ladder = pd.read_csv(DATA_DIR + 'Ladder_peaks.csv')
    """
    Exclude small peaks from the electropherogram, which do not correspond to actual markers in the ladder.
    """
    ladder_ticks = peak_ladder.query("Peak_Height >= 1")
    """
    Place the ladder ticks at the correct times on the x-axis.
    """
    peaks = list(ladder_ticks['Aligned Migration Time [s]'])
    ax.set_yticks([])
    ax.set_ylim(0, 5)
    plt.xticks(ticks=peaks[3:-1], labels=[1000, 2000, 4000], fontsize=14)
    sns.despine()
    plt.tight_layout()
    plt.show()
  e. The resulting plot is reported in Figure 3B in expected outcomes.
29. As the concentration of the mRNA dilutes throughout the different peaks for increasing PABP concentrations, normalizing the data to the highest peak of the sample, instead of the marker, might help to enhance the peaks at high PABP concentrations. To do this:
  a. Identify the max value in the peaks of the control sample (0 μM PABP):
    """
    Specify the control sample (i.e. the one with 0 μM PABP)
    """
    control = 0
    No_PABP = pd.read_csv(DATA_DIR+'Sample_'+str(control)+'.csv',
           skiprows=16,
           skipfooter=1,
           engine='python')
    """
    Specify in which range of seconds the sample peaks occur and get the max value Make sure to exclude the marker peak from this range
    """
    No_PABP = No_PABP.query('31.5 <= Time <= 42')
    ref_peak = No_PABP.Value.max()
    """
    Normalize all the peaks to the identified max value
    """
    No_PABP["Value_norm"] = Normalize_to_ref(No_PABP.Value, ref_peak)
    ax.plot(No_PABP.Time, No_PABP.Value_norm, color='k', lw=3, alpha=1)
  b. Do the same with each sample containing PABP and plot with vertical shift.
    """
    Define the vertical shift
    """
    move = 0.3
    for file_nr, name in file_dir.items():
     """
     Skip control sample, since that is already plotted
     """
     if file_nr == control:
      continue
     electrogram =
     pd.read_csv(DATA_DIR+'Sample_'+str(file_nr)+'.csv',
               skiprows=16,
               skipfooter=1,
               engine='python')
    """
    Find the marker peak of each sample
    """
    electrogram_norm = electrogram.query('31.5 <= Time <= 42')
    ref_peak = electrogram_norm.Value.max()
    electrogram = electrogram.query('31.5 <= Time <= 42')
    electrogram["Value_norm"] =
    Normalize_to_ref(electrogram.Value, ref_peak)
    ax.plot(electrogram.Time, electrogram.Value_norm + move,
    color=palette[file_nr], lw=3)
    move += 0.3
  c. Annotate and show the plot as explained in steps 25c and 25d.
  d. The resulting plot is reported in Figure 3C in expected outcomes.

## Expected outcomes

The successful synthesis of PCR products with increasing poly(A)-tail length is visible in a non-denaturing agarose gel, showing a single band for each lane, and bands shift upwards with increasing tail lengths (Figure 1A). The sequencing reactions of the tailed PCR products should show a sequence consisting of repeating A nucleotides (Figure 1B), of a length close to the expected one (i.e., the one used in the tailed primers).

The *in vitro* synthesized mRNAs should show a single narrow peak when analyzed via capillary electrophoresis, corresponding to the expected length of the poly(A)-tailed mRNA (Figure 2A). Similar to the PCR products, the gel-like image (or the non-denaturing agarose gel) should have a single clear band for each lane, and bands shift upwards with increasing tail lengths (Figure 2B). The sequencing of the tailed mRNA products should show a sequence consisting of only A nucleotides (Figure 2C).

The mobility shift assay should show a single band for the 0 μM PABP sample, with periodic appearance of peaks at larger sizes for increasing PABP concentrations (Figure 3A). It is essential to note that since the same concentration of RNA is loaded in each lane, the appearance of new peaks leads to a redistribution of the mRNAs across multiple bands of lower concentrations. This results in the formation of dimer bands (Figure 3B) and increased noise (highlighted by the red box in Figure 3A). Consequently, the signal-to-noise ratio might become lower as the mRNA signal decreases. For this reason, to better visualize and display the results, we suggest normalizing the peaks to the highest peak value of the sample (excluding the marker), as explained in step 26 and shown in Figure 3C. As the poly(A)-tail length increases, new peaks should appear at higher molecular weights (Figures 3C and 3D).

## Limitations

Although we have demonstrated accurate synthesis of poly(A)-tailed mRNAs within the range of 5 to 150 nucleotides, extending the poly(A)-tail length beyond 150 nucleotides may introduce non-specific products and impurities at both the DNA and mRNA levels. Additionally, the sensitivity of the mobility shift assay may vary depending on factors such as RNA quality, concentration, and experimental conditions, potentially impacting the detection of low-abundance mRNA species. While the *in vitro* approach provides a controlled environment for studying PABPC binding, it may not fully recapitulate the cellular context, potentially resulting in differences in binding kinetics or protein interactions. The application of the same protocol to other mRNA sequences, RBPs or biological contexts may necessitate further validation.

## Troubleshooting

### Problem 1

The PCR products do not show clear bands for the long poly(A)-tails (related to step 6).

### Potential solution

• Try using an untailed linear DNA template instead of the circularized plasmid, or use a shorter tailed PCR product as template (e.g., use the 50 nt tailed PCR product as template for the 100 nt tailed PCR product).
• Lowering the amount of PCR cycles could help decrease the appearance of unspecific products.
• When using long tailed reverse primers (>100 nt), these could be retained even after purification of the PCR products, and appear as small bands at the end of the gel. These should not affect the IVT reaction, but if preferred they can be removed by purifying the products of interest from the agarose gel.

### Problem 2

The poly(A)-tail sequence contains a lot of non-A nucleotides (related to step 15).

### Potential solution

• DNA polymerase: if the problem appears to be on the PCR product level, try testing different DNA polymerases for template amplification, as fidelity varies among different polymerases.
• RNA polymerase: similar to the DNA polymerases, different RNA polymerases can have different fidelities. Therefore, testing different mutants or types could help obtain the desired products.

### Problem 3

The IVT yield is very low (related to steps 8–13).

### Potential solution

• Template concentration: we suggest testing a range of template concentrations and determine which one that gives the highest yield.
• Incubation time: depending on the length of your template you might need to increase the incubation time of the IVT reaction.
• 5′UTR length: we have noticed that increasing the sequence length upstream of the T7 promoter can help increasing transcription efficiency, probably by facilitating a better binding of the polymerase to the promoter.

### Problem 4

No PCR product after the amplification of the linker-ligated mRNAs (relates to steps 15a–15n).

### Potential solution

The reason why you do not have any PCR product after the amplification of the linker-ligated mRNAs could be related to an unsuccessful ligation reaction. T4 RNA ligase 1 has a certain sequence preference and does not ligate all RNA ends with the same efficiency. If this is the case, you can switch to ligation with T4 RNA ligase 2, truncated K227Q and a universal preadenylated miRNA cloning linker (NEB). Alternatively, optimization of the PCR annealing temperature, the number of PCR cycles or using a different primer sequence might result in higher and/or cleaner PCR product yield.

### Problem 5

The shift mobility assay does not show clear peaks appearing (related to steps 16–18).

### Potential solution

• If the size of the mRNA transcript is very big (>2000 nt), the binding of the RBP might create a ribonucleoprotein (RNP) complex too big to enter the gel. One solution could be synthesizing a shorter mRNA transcript containing only the sequence of interest for the binding.
• Another cause could be related to the conditions used for the binding reaction. A solution to this problem can be testing different mRNA and protein concentrations to the ones suggested by us. Also varying the salt concentrations might influence protein/RNA binding.
• Ensure the RNA is of high purity. If the RNA exhibits non-specific peaks or broad peaks prior to the *in vitro* binding assay, it can be difficult to distinguish between different numbers of bound proteins. Improve the RNA purification process to achieve clearer results.

## Resource availability

### Lead contact

Further information and requests for resources and reagents should be directed to and will be fulfilled by the lead contact, Maike M. K. Hansen (maike.hansen@ru.nl).

### Technical contact

Questions about the technical specifics of performing the protocol should be directed to and will be answered by the technical contact, Carmen Grandi (carmen.grandi@ru.nl).

### Materials availability

This study did not generate new materials.

### Data and code availability

The accession number for the code generated and the reference dataset reported in this paper is https://doi.org/10.5281/zenodo.13364571. Hansen-Labs/ShiftMobilityAssay (github.com

## Acknowledgments

We thank the generous support from 10.13039/501100001832Radboud University, the Christine Mohrmann fellowship, the 10.13039/501100003246Netherlands Organization for Scientific Research (NWO, VI.Vidi.223.065), and 10.13039/501100021821Oncode Institute, which is partly financed by the Dutch Cancer Society.

## Author contributions

C.G., M.E., and M.M.K.H. conceived and designed the study. C.G., M.E., and F.H.T.N. performed the experiments. C.G. wrote the manuscript. M.M.K.H., M.E., and F.H.T.N. edited the manuscript.

## Declaration of interests

The authors declare no competing interests.
